# Imaging of tumour response to immunotherapy

**DOI:** 10.1186/s41747-019-0134-1

**Published:** 2020-01-03

**Authors:** Clarisse Dromain, Catherine Beigelman, Chiara Pozzessere, Rafael Duran, Antonia Digklia

**Affiliations:** 1 Department of Radiology and Interventional Radiology, Lausanne University Hospital and University of Lausanne, Rue du Bugnon 46, CH-1011 Lausanne, Switzerland; 2 Department of Radiology, AUSL Toscana Centro - San Giuseppe Hospital, Empoli, Italy; 3 Department of Oncology, Lausanne University Hospital and University of Lausanne, Lausanne, Switzerland

**Keywords:** Cell- and tissue-based therapy, Immunotherapy, Immune checkpoint inhibitors, Pseudoprogression, Response evaluation criteria in solid tumors (RECIST)

## Abstract

A wide range of cancer immunotherapy approaches has been developed including non-specific immune-stimulants such as cytokines, cancer vaccines, immune checkpoint inhibitors (ICIs), and adoptive T cell therapy. Among them, ICIs are the most commonly used and intensively studied. Since 2011, these drugs have received marketing authorisation for melanoma, lung, bladder, renal, and head and neck cancers, with remarkable and long-lasting treatment response in some patients. The novel mechanism of action of ICIs, with immune and T cell activation, leads to unusual patterns of response on imaging, with the advent of so-called pseudoprogression being more pronounced and frequently observed when compared to other anticancer therapies. Pseudoprogression, described in about 2–10% of patients treated with ICIs, corresponds to an increase of tumour burden and/or the appearance of new lesions due to infiltration by activated T cells before the disease responds to therapy. To overcome the limitation of response evaluation criteria in solid tumors (RECIST) to assess these specific changes, new imaging criteria—so-called immune-related response criteria and then immune-related RECIST (irRECIST)—were proposed. The major modification involved the inclusion of the measurements of new target lesions into disease assessments and the need for a 4-week re-assessment to confirm or not confirm progression. The RECIST working group introduced the new concept of “unconfirmed progression”, into the irRECIST. This paper reviews current immunotherapeutic approaches and summarises radiologic criteria to evaluate new patterns of response to immunotherapy. Furthermore, imaging features of immunotherapy-related adverse events and available predictive biomarkers of response are presented.

## Key points

- Immune checkpoint inhibitors remove inhibitory signals of T cell activation
- Pseudoprogression occurs in 2–10% of patients treated with immunotherapy
- An increase of tumour burden during immune checkpoint inhibitor treatment is more likely to reflect true progression than pseudo-progression
- New criteria to assess immunotherapy are based on two major assumptions: new lesions do not preclude a progressive disease and a progression need to be confirmed on 4–8 weeks follow-up imaging
- The knowledge of immune-related adverse events is of utmost importance and requires the exclusion of differentials, mainly of infectious or tumour nature

## Background

Cancer immune surveillance plays an important role in the origin and pathogenesis of cancer. Three essential phases, *i.e.,* elimination, equilibrium, and escape, appear to contribute to tumourigenesis and tumour progression [1]. This dynamic crosstalk between tumour and immune system is crucial. Over recent years, the identification of key players of this interaction has led to an immense breakthrough in cancer therapeutics with development of new anticancer drugs targeting the immune system instead of the tumour cells.

Patterns of disease response, stability, and progression to immunotherapy may differ from those observed with other drugs, such as chemotherapies and targeted therapies. Indeed, some patients experience a response after an initial progression, so-called *pseudoprogression*, that has led to the development of immune-specific related response criteria where treatment may be used beyond a progression evaluated according to the “response evaluation criteria in solid tumors” (RECIST) criteria [2].

Although immune-checkpoint inhibitors (ICIs) are safer compared to cytotoxic chemotherapy, various specific immunotherapy-related adverse events (irAEs) can be often detected on imaging, even before the onset of symptoms. Their prompt identification, systematically requiring to exclude differentials, is crucial to allow an optimal management.

In this paper, we aim to review the different approaches of immunotherapy and the specific patterns of disease response and progression to these new drugs, especially to ICIs. Then, we describe the new criteria developed to assess response to immunotherapy and discuss the major immune-related side effects.

### Different immunotherapy approaches

**Fig. 1:**
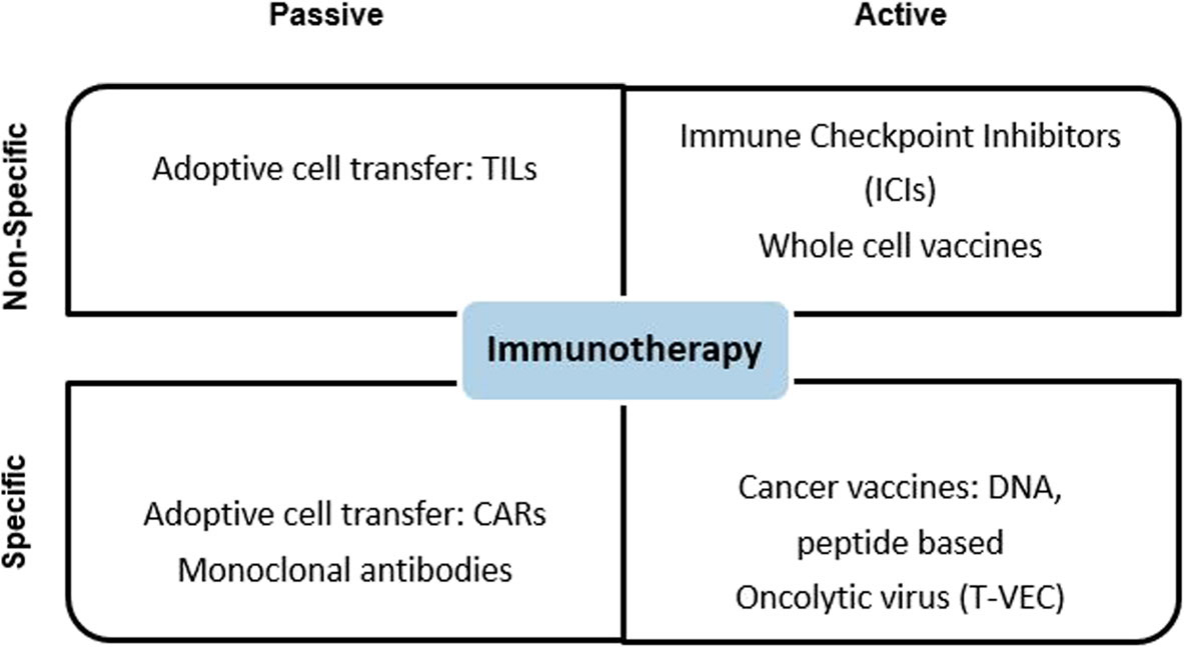
Different approaches of immunotherapy. *CAR* Chimeric antigen receptor, *DNA* Deoxyribonucleic acid, *TILs* Tumour-infiltrating lymphocytes, *T-VEC* Talimogene laherparepvec

There are different types of immunotherapy (Fig. 1). Some of these are non-specific immunotherapy, such as ICIs, leading to a general stimulation of the immune system, whereas others are more tumour-specific. Tumour-specific immunotherapy is based on the recognition by immune cells of unique tumour-specific antigens and includes different types of therapeutic approaches such as oncolytic virus, cancer vaccines, and adoptive cell transfer. Among these emerging approaches of immunotherapy, the ICIs—anti-programmed cell death protein 1 (PD-1), anti-programmed cell death protein ligand 1 (PD-L1), and anti-cytotoxic T-lymphocyte antigen (CTLA4)—are the most thoroughly investigated class of immunotherapy and increasingly used in routine clinical practice.

#### Oncolytic viruses

The oncolytic viruses hold great promise in the fight against cancer since it is designed to work by selective replication in cancer cells and to cause their death through several mechanisms including promotion of cellular immunity and hijacking of cellular death pathways [3].

Several types of parental viruses are used including herpes simplex virus type 1 and adenoviruses. Talimogene laherparepvec (Imlygic™) consists of an engineered, genetically modified herpes simplex virus type 1. It can infect and selectively destroy malignant cells while activating the immune system by the coding sequence of the granulocyte-macrophage colony-stimulating factor for immunostimulation. This virus demonstrated to be immunogenic and safe for the local treatment of unresectable cutaneous, subcutaneous, and nodal lesions in patients with recurrent melanoma after primary surgery. It is currently approved for this indication in several countries and was approved by the US Food and Drug Administration (FDA) and the European Medicines Agency [4, 5].

Approximately half of the patients had symptoms of fatigue and chills/fever during the treatment, and roughly a third of them had flu-like symptoms and nausea. There were also some rare but serious side effects including cellulitis, vitiligo, deep vein thrombosis, vasculitis, herpes virus infection, and herpes simplex keratitis [4]. Several clinical trials evaluating the intratumoural injection of talimogene laherparepvec or other oncolytic viruses (*e.g.,* intrahepatic, intrapancreatic, intraprostatic, or into breast lesions) alone or in combination with ICIs are ongoing.

#### Cancer vaccines

T cells are characterised by the expression of T cell receptors capable of recognising intracellular antigenic peptides uniquely expressed on the surface of major histocompatibility complex molecules. The recognition of foreign antigens such as viral proteins or altered antigens such as the products of mutated cancer genes by T cell receptors leads to their activation.

Currently, many diverse therapeutic vaccination strategies are being developed or evaluated in clinical trials including cell vaccines (autologous or allogeneic tumour or immune cell), protein/peptide vaccines, and genetic—deoxyribonucleic acid (DNA), ribonucleic acid (RNA), and viral—vaccines depending on the sources of the antigens [6]. A promising approach is the use of the most potent antigen-presenting cells, the so-called circulating dendritic cells, based on their capacity to initiate and directly modulate specific immune responses [7].

In this context, naturally circulating dendritic cells are isolated by leukapheresis (see below) and then loaded *ex vivo* with tumour antigens. Then, they are intravenous-administered into cancer patients to induce tumour-specific effector T cells aimed at recognising and eliminating cancer cells as well as inducing immunological memory to control tumour growth [6, 8].

#### Adoptive cell transfer

This treatment is based on the intravenous infusion of tumour-specific T cells. These cells can be isolated from one of two sources: (i) autologous tumour-infiltrating lymphocytes (TILs) from the tumour mass and (ii) autologous T cells isolated from patient’s peripheral blood (leukapheresis) that have been genetically modified to express chimeric antigen receptors or specific anti-tumour T cell receptors reactive to specific tumour-associated antigens [11]. Exposition to high dose of interleukine-2 *ex vivo* leads to their activation and expansion before being re-infused to patients after lympho-depleting chemotherapy.

Autologous TIL therapy has been used for more than 10 years in melanoma patients and has resulted in durable and in some cases complete response [9]. Regarding solid tumours, TIL therapy is limited by the low availability and infiltration of TILs in tumour mass as well as their exhaustion. Several studies are ongoing to improve methods of *ex vivo* expansion and their reconditioning. Chimeric antigen receptor T cell has already been considered as a breakthrough in haematological cancers, with two drugs targeting antigen CD19, tisagenlecleucel (Kymriah™) and axicabtagene ciloleucel (Yescarta™), that were FDA-approved in 2018 for B cell lymphomas and leukaemias [10, 11].

Despite looking very promising, these sophisticated approaches have severe toxicities that can be life-threatening or fatal. These toxicities include the cytokine release syndrome, consisting of high fever and flu-like symptoms, hypotension, and pulmonary fluid overload, as well as neurotoxicity and capillary leak syndrome that require to be managed under close observation. Therefore, this type of treatment is administered only at certified centres.

#### Immune checkpoint inhibitors

ICIs are a new class of cancer immunotherapy drugs that act as negative regulators of multiple immune checkpoints, particularly in cytotoxic T cells, leading to inhibition of T cell stimulation. The negative costimulatory molecules such as CTLA-4, PD-1, T cell immunoglobulin, and mucin domain-3 and lymphocyte-associated gene 3 are expressed in different immune cell types, including cytotoxic T cells, B cells, natural killer cells, monocytes, tumour-associated macrophages, myeloid-derived suppressor cells and dendritic cells exhibiting immunosuppressive functions. As a result, T cells are exhausted and the anti-cancer functions of the immune system are weakened. ICIs remove these inhibitory signals, restore T cells from their exhausted status, and recover their cytotoxicity on tumour cells. Although rescue of exhausted T cells or depletion of regulatory T cells is the primary function of ICIs, regulation of T cell trafficking and migration have been also reported [12]. However, it was not until 2011 that the ICI ipilimumab (Yervoy™), an anti-CTLA-4 monoclonal antibody, was approved for metastatic melanoma, followed by the development of other drugs such as PD-1 and PD-L1 inhibitors. Currently, seven ICIs are about to be FDA-approved for a range of indications, in monotherapy or in combination with other drugs. They consist of one anti-CTLA-4 (ipilimumab (Yervoy®)), three anti-PD-1 (pembrolizumab (Keytruda®), nivolumab (Opdivo®), and cemiplimab (Libtayo®)), and three anti-PD-L1 (atezolizumab (*Tecentriq*®), durvalumab (Imfinzi®), and avelumab (*Bavencio*®)) (Tables 1 and 2) [13].

**Table 1:** Clinical indications of the different immune checkpoint inhibitors

Immune checkpoint inhibitor	Target	Indications
Ipilimumab	CTLA-4	Colorectal cancer, metastatic (microsatellite instability-high or mismatch repair deficient in combination with nivolumab) Melanoma, unresectable, or metastatic in combination with nivolumab Melanoma, adjuvant treatment Advanced renal cell cancer, in combination with nivolumab
Pembrolizumab	PD-1	Recurrent or metastatic cervical cancer Advanced or metastatic gastric cancer Head and neck cancer, squamous cell, unresectable/recurrent or metastatic, alone or in combination with chemotherapy Advanced hepatocellular carcinoma Hodgkin lymphoma, classical, relapsed or refractory Melanoma, adjuvant treatment Melanoma, unresectable or metastatic Merkel cell carcinoma, recurrent or metastatic Microsatellite instability-high cancer, unresectable or metastatic NSCLC, stage III or metastatic, single-agent therapy NSCLC, metastatic, non-squamous, combination therapy with chemotherapy Primary mediastinal large B cell lymphoma, relapsed or refractory Advanced renal cell carcinoma Small cell lung cancer, metastatic Urothelial carcinoma, locally advanced or metastatic
Nivolumab	PD-1	Like pembrolizumab
Cemiplimab	PD-1	Cutaneous squamous cell carcinoma, metastatic or locally advanced
Atezolizumab	PD-L1	Breast cancer (triple-negative), locally advanced or metastatic in combination with nab-paclitaxel NSCLC, metastatic: first line with bevacizumab, paclitaxel, and carboplatin Previously-treated NSCLC: monotherapy Small cell lung cancer, extensive-stage: first-line treatment with carboplatin and etoposide Urothelial carcinoma, locally advanced or metastatic
Durvalumab	PD-L1	NSCLC (stage III), unresectable, initiated within 6 weeks after chemo-radiotherapy Urothelial carcinoma, locally advanced or metastatic
Avelumab	PD-L1	Metastatic Merkel cell carcinoma Advanced renal cell carcinoma, in combination with axitinib Urothelial carcinoma, locally advanced or metastatic

*CTLA4* Cytotoxic T-lymphocyte antigen 4, *NSCLC* Non-small cell lung cancer, *PD-1* Programmed cell death protein 1, *PD-L1* Programmed cell death protein ligand 1

**Table 2:** Rate of pseudoprogression in patients with melanoma or NSCLC

First author, year [reference]	Number of patients	Type of cancer	Treatment	Pseudoprogression (%)
Wolchock, 2009 [17]	227	Melanoma	Ipilimumab	9.7
Hodi, 2016 [26]	327	Melanoma	Pembrolizumab	7.0
Nishino, 2017 [24]	107	Melanoma	Pembrolizumab	5.0
Gettinger, 2015 [84]	129	NSCLC	Nivolumab	5.0
Nishino, 2017 [85]	160	NSCLC	Nivolumab or pembrolizumab	0.6
Katz, 2018 [86]	166	NSCLC	Anti-PD1 (nivolumab 80%)	2.0
Fujimoto, 2019 [27]	542	NSCLC	Nivolumab	3.0

*PD-1* Programmed cell death protein 1, *NSCLC* Non-small cell lung cancer

### How to assess response to treatment

#### Characteristics of response

Responses obtained after ICI immunotherapy are different from those observed after cytotoxic chemotherapy. Although chemotherapy has a transient effect with reduced tumour growth kinetic only during its administration and re-growth after discontinuation, immunotherapy may alter the biology of the patient by inducing a memory cell response which includes memory T cells that may provide long-term immune protection [14–16].

Responses to immunotherapy have been described to be more delayed with slower decrease of the total tumour burden but with durable response even after stopping the treatment [17, 18]. Although ICIs work only in a subgroup or a minority of patients, they can induce durable responses in 10–20% of treated patients, even after the discontinuation of treatment, providing a survival benefit [19, 20]. For example, for the first time in melanoma history, ICIs induce long-lasting remission exceeding 5 years [21].

**Fig. 2:**
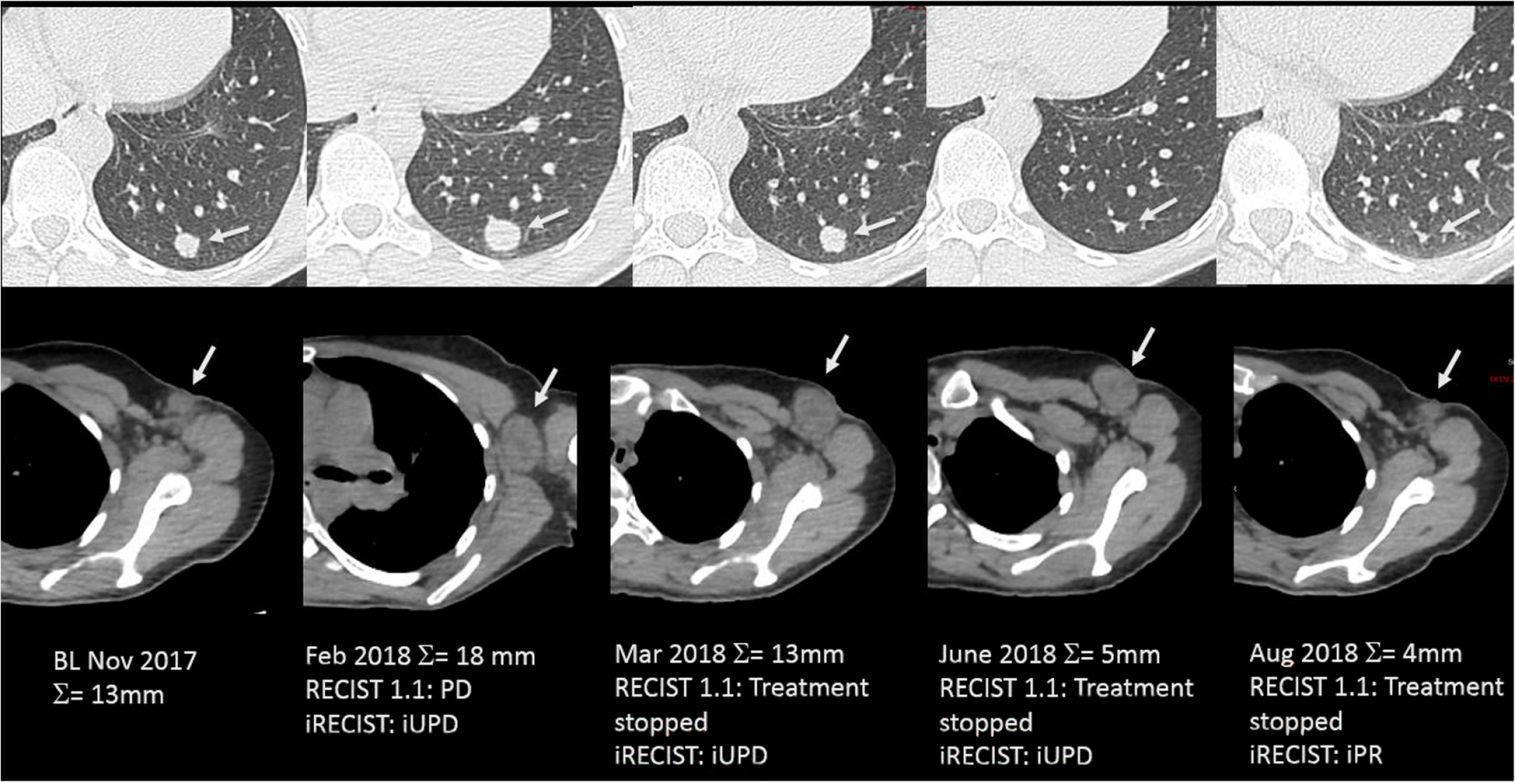
Comparison of RECIST 1.1 and iRECIST criteria for evaluation of a 45-year-old woman with metastatic melanoma treated with ipilimumab (antiCTLA-4) and nivolumab (anti-PD-1). Baseline CT image (November 2017) shows a 13-mm lung metastasis (target lesion, upper panel, arrow) and a 10-mm short-axis axillary lymph node (non-target lesion, lower panel, arrow). On a 3-month follow-up, both lesions enlarged with an increase of 38% of the target lesion leading to a progressive disease (PD) and a stop of treatment according to RECIST 1.1 and an unconfirmed PD with maintained treatment according to iRECIST criteria. On the two following CT examinations (March and June 2018), the lung metastasis decreased in size, but the axillary lymph node was stable (unconfirmed PD) but still significantly enlarged compared to the baseline (still unconfirmed PD according to iRECIST criteria). Finally, on August 2018, CT images showed a decrease in size of both lesions, confirming the pseudoprogression with a response assessed to be -70%, leading to a partial response according to iRECIST criteria

**Fig. 3:**
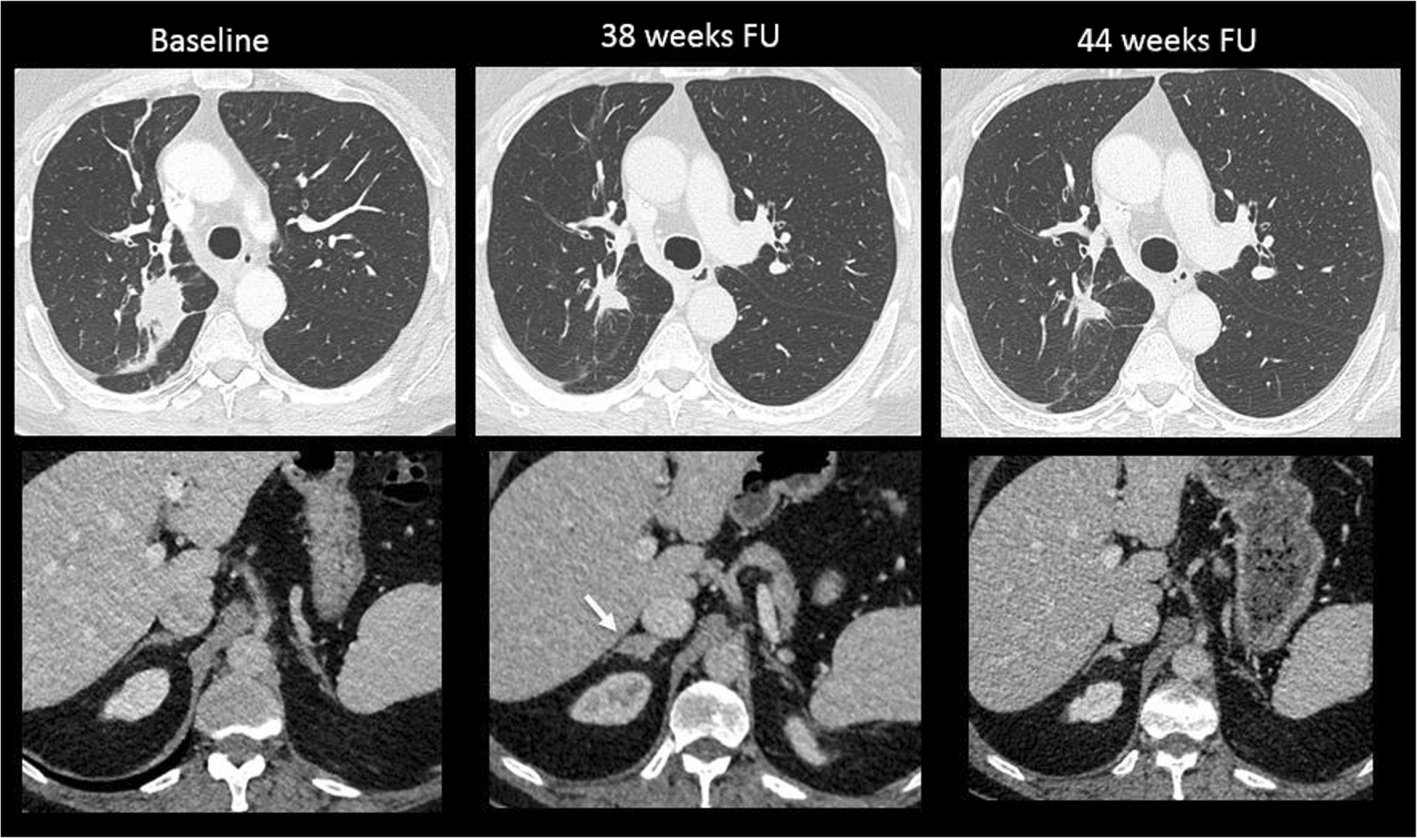
Pseudoprogression in a 65-year-old patient with lung carcinoma treated with nivolumab (anti-PD-1). Baseline axial CT showed a lung mass in the upper right lobe with normal adrenal glands. At a 38-week follow-up (FU), there was a good reduction in the size of the lung mass, but a new lesion appeared in the right adrenal gland (arrow). The patient was maintained under the same treatment. At 44-week follow-up, the right adrenal mass disappeared, confirming the diagnosis of pseudoprogression

Moreover, two new forms of response patterns, so-called pseudoprogressions, were observed initially in patients with advanced melanoma treated with ipilimumab [17]:

- A response after an initial increase in total tumour volume (Fig. 2)
- A reduction in total tumour burden after the appearance of new lesions (Fig. 3)

Pseudoprogression does not reflect tumour cell growth but may be misclassified as progressive disease. The mechanism behind pseudoprogression could be related to the infiltration of T cells into tumours, resulting initially in an apparent increase in tumour burden rather than true proliferation of tumour cells [17]. Associated inflammatory reaction, due to cytokine release, has been also observed in on-treatment biopsy samples performed after radiological progression in patients treated with ipilimumab [22]. Another explanation could be the time required to mount an adaptive immune response resulting in a continued tumour growth until a sufficient response develops [23].

Although pseudoprogression in patients treated with ICIs has been hotly debated, its incidence is actually low and differs depending on the tumour type (for example, less frequent in non-small cell lung cancer patients (< 5%) than in melanoma (< 10%)) (Table 2). As a consequence, an increase of tumour burden during ICI treatment is more likely to reflect true progression rather than pseudoprogression.

Pseudoprogression has been found to be more frequent in younger patients, probably because of the better reactivity of the immune system, and may occur at any time after the onset of therapy [24]. Pseudoprogression was mostly observed around 12 weeks, in particular in melanoma patients treated with ipilimumab, although more delayed pseudoprogression was also reported [25]. In melanoma patients, it has been shown that this phenomenon can occur in lymph nodes, but more commonly in non-nodal locations such as the kidneys, liver, lungs, peritoneum, adrenal glands, and chest and abdominal walls [26]. Finally, patients experiencing a pseudoprogression have been shown to have a shorter duration of response than patients with a typical response, but a better chance of survival than patients with typical progression [27].

**Fig. 4:**
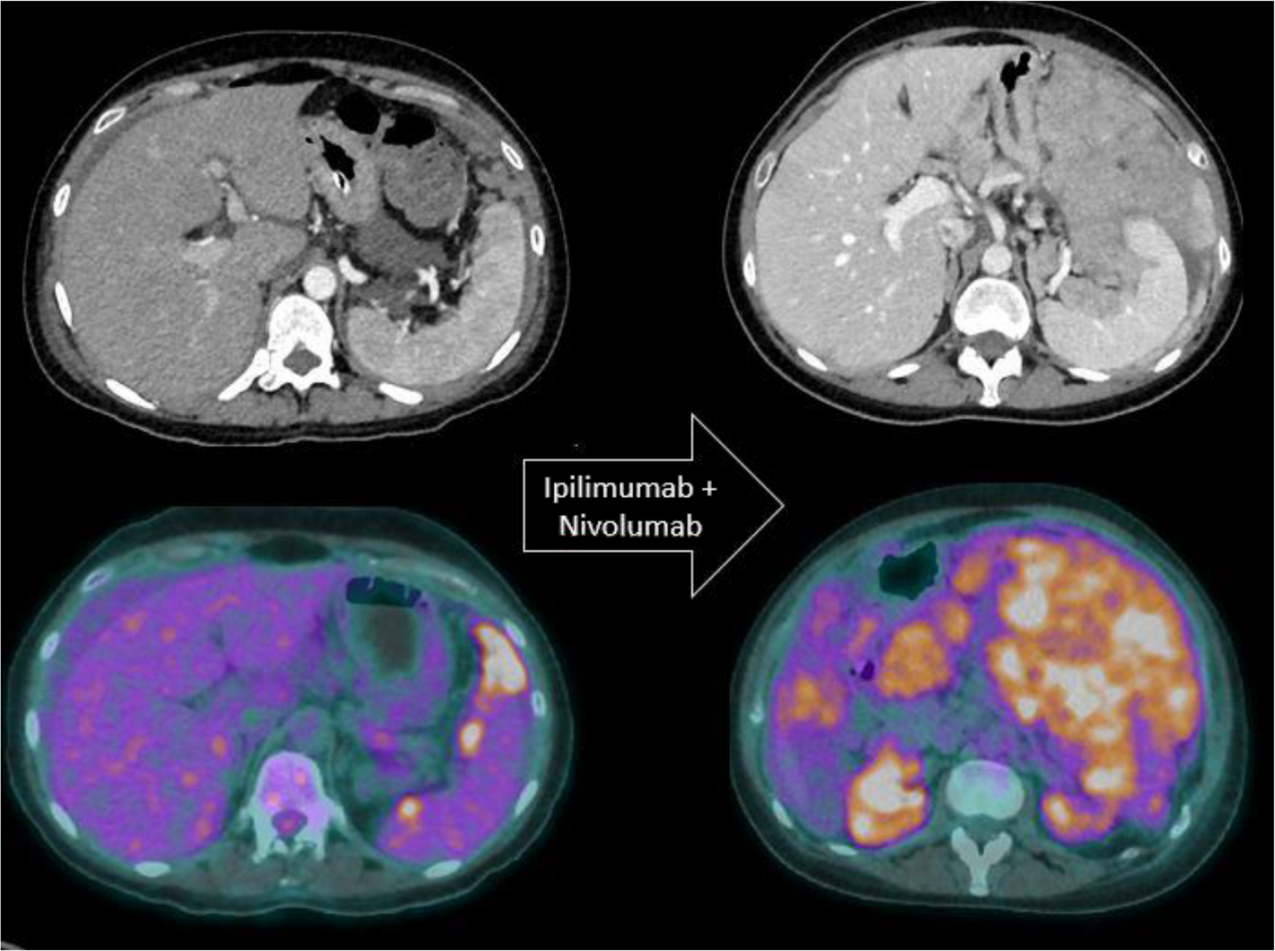
Paradoxical acceleration of tumour growth kinetics in a patient with metastatic melanoma treated with ipilimumab and nivolumab. Baseline axial CT image and corresponding ^18^F-FDG PET/CT image show few perisplenic peritoneal metastatic implants. Two months after the initiation of immunotherapy, both imaging modalities show a dramatic increase in peritoneal metastases.

Another atypical response after initiation of immunotherapy is the hyperprogression, *i.e.* a paradoxical acceleration of tumour growth kinetics. It has been described after the onset of anti-PD1/PD-L1 therapy with an incidence of about 10% [28] (Fig. 4). To avoid misdiagnosing treatment-related disease hyperprogression with conventional progressive disease, it has been suggested to use the tumour growth rate to compare the growth rate before and after the initiation of treatment [29]. Using a definition of ≥ 2-fold increase of tumour growth rate before and after anti-PD-1/PD-L1 therapy, a hyperprogressive disease was found in 12 of 218 patients (9%) [29]. No association was found between hyperprogression and baseline tumour burden, the type of the immunotherapy, tumour histology, and number of previous lines of treatment. Nevertheless, hyperprogression was significantly correlated with patients’ age and decreased overall survival.

Notwithstanding these findings, the attribution of hyperprogression to immunotherapy remains controversial. In particular, hyperprogression has been observed in patients having received other therapies, such as surgery, radiotherapy, and/or chemotherapy or even in the absence of treatment [30, 31]. Moreover, the mechanisms underlying hyperprogressive disease have not been elucidated yet.

#### New criteria to assess the response to immunotherapy

Although relatively infrequent, these atypical response patterns have important implications for patient management. To address the issue of pseudoprogression and provide standardisation for assessing response to immunotherapy, new criteria have been developed. All these criteria are based on two major statements: (1) new lesions do not preclude progressive disease and (2) a confirmation of progressive disease is required.

Immune-related response criteria (irRC) were developed for melanoma treated with ipilimumab and based on modified World Health Organization criteria, which use bi-dimensional tumour measurements (five lesions per organ, up to ten visceral lesions and five cutaneous index lesions) burden [17, 32]. The major differences compared to the World Health Organization and RECIST criteria were the incorporation of measurable new lesions into the total tumour burden [2, 17] (Table 3). Moreover, response was allowed after an initial progression. Consequently, complete response is defined as disappearance of all target lesions, partial response as a ≥ 50% reduction in the sum of target lesions, stable disease as neither sufficient shrinkage to qualify for partial response nor sufficient increase to qualify for progressive disease, and progressive disease as ≥ 25% increase of the sum of target lesions plus new measurable lesion in comparison with the disease nadir.

**Table 3:** Comparison of the different criteria developed for the assessment of response to immunotherapy

Criteria, year [reference]	irRC, 2009 [17]	irRECIST, 2013 [33]	iRECIST, 2017 [34]	imRECIST, 2018 [35]
Baseline				
Definition of target lesion	World Health Organization criteria +5 cutaneous targets	RECIST 1.1	RECIST 1.1	RECIST 1.1
Definition of non-target lesion	Not specified	RECIST 1.1	RECIST 1.1	RECIST 1.1
Definition of lymph node	Not specified	RECIST 1.1	RECIST 1.1	RECIST 1.1
Follow-up				
New lesion	≥ 5 × 5 mm; up to 5/organ; 5 new cutaneous and 10 visceral lesions PD not defined Measurement of new lesions included in the total tumour burden	RECIST 1.1 PD not defined Measurement of new lesions included in the total tumour burden	RECIST 1.1 Defined unconfirmed PD	RECIST 1.1 PD not defined
Non-target lesion	Only to define irCR	Only to define irCR	RECIST 1.1 May define UPD	Only to define irCR
PD definition	Determined only on measurable disease (≥ 25% increase in the sum of target lesions and new lesions from the nadir) Negated by subsequent non-PD assessment ≥ 4 weeks	Determined only on measurable disease (≥ 20% increase in the sum of target lesions and new lesions from the nadir) Negated by subsequent non-PD assessment ≥ 4 weeks	Confirmed PD if : - Unconfirmed PD of target lesions on previous exam and increase in tumour burden of target lesions ≥ 5 mm - Unconfirmed PD of non-target lesions and their significant increase - Unconfirmed PD for new lesions and increase in tumour burden ≥ 5 mm or increase in the number of new lesion	Determined only on measurable disease (≥ 20% increase in the sum of target lesion and new lesions from the nadir) The presence of new lesions does not define PD Negated by subsequent non-PD assessment ≥ 4 weeks

*irRC* Immune-related response criteria, *imRECIST* Immune-modified RECIST, *PD* Progressive disease

The immune-related response criteria using unidimensional measurement, so-called irRECIST, has been developed based on RECIST 1.1 [2] adaptation of the irRC [33]. These new criteria have been found to provide a higher reproducibility compared to irRC, to be more practical in clinical routine, and to provide response assessment that can be directly compared to the results from other clinical trials based on RECIST 1.1 criteria [2]. Due to the unidimensional measurement, partial response is now defined as ≥ 30% reduction in the sum of target lesions, stable disease as sum of target lesions < 20% increase and < 30% reduction, and a progressive disease as ≥ 20% increase of the sum of target lesions plus new measurable lesions from the nadir.

More recently, the RECIST working group published the modified RECIST 1.1 for immune-based therapy, so-called immune-related RECIST (irRECIST) [34]. Also using RECIST 1.1-based measurement, irRECIST introduced the new concept of “unconfirmed progressive disease” corresponding to progressive disease that remains to be confirmed on a 4–8-week follow-up imaging. If the patient condition is classified as unconfirmed progressive disease and is clinically stable, treatment should be continued. As opposed to other criteria developed for immunotherapy, a non-target lesion progression can define a progressive disease. Following irRECIST criteria, a partial response is defined as a ≥ 30% reduction in the sum of target lesions and unconfirmed progressive disease as ≥ 20% increase in the sum of target lesions from the nadir or non-target lesion progression or appearance of new lesion.

The progressive disease is confirmed in case of:

- Target lesions previously classified as unconfirmed progressive disease and presence of an increase in tumour burden of target lesions ≥ 5 mm on the 4–8-week follow-up imaging;
- Or non-target lesion previously classified as unconfirmed progressive disease and significant increase of non-target lesion on the 4–8-week follow-up imaging;
- Or new lesions resulting of UPD and increase of tumour burden ≥ 5 mm of these new lesions or increase in the number of new lesions on the 4–8-week follow-up imaging.

For progression-free survival assessment, the date used for progressive disease is the first unconfirmed progressive disease date, if the latter is subsequently confirmed.

Finally, the immune-modified RECIST (imRECIST) criteria were developed initially for implementation of atezolizumab studies [35]. These criteria include key principles of irRC applied with unidimentional RECIST 1.1 criteria, similarly to irRECIST criteria and share the same definition of response and progression as the irRECIST.

The differences between these criteria are summarised in Table 3. In clinical trials, irRECIST and imRECIST are the most promising criteria to assess response rate and progression-free survival that are the most commonly used surrogate endpoints to assess overall survival [36]. However, data are still limited, in particular in other types of cancers than melanoma and non-small cell lung cancer, to draw any definitive conclusion. Accordingly, these criteria, developed for clinical trial, should be used with caution in clinical routine.

### Immune-related adverse events: the role of imaging

Although ICIs are safer compared to cytotoxic chemotherapy, they enhance the immune activity and may cause a dysregulation of immune homeostasis in normal tissues, which may lead to specific toxicities called “immune-related adverse events” (irAEs). They generally start the first few weeks after treatment; nevertheless, they can occur at any time, even after treatment discontinuation [37–39]. Their incidence and severity depend on the agent, with higher all-grade rates reported with anti-CTLA4 (up to 80%) compared to anti-PD1 (27%) and anti-PDL1 (17%) [39–42]. Different tissues and organs may be affected and multisystem toxicities are common, with a spectrum of imaging manifestations in each organ [43]. Fatigue, cutaneous toxicities, colitis, and endocrine dysfunctions are the most frequent events, followed by hepatitis and pneumonitis [39–42]. Other rare irAEs include nephrologic, neurologic, cardiologic, and haematologic toxicities [37, 40–42]. Although they are generally manageable mild toxicities, severe and life-threatening events may occur in up to 7%, 3%, and 30% of patients receiving anti-PD1, anti-PDL1, and anti-CTLA4, respectively, reaching up to 55% with combined immunotherapy [39–42]. IrAEs generally respond to immunotherapy holding and require corticosteroids and immunosuppressive treatment in more severe cases along with organ-specific treatment. Radiologic manifestations of irAEs can be found in up to 17% of patients receiving immunotherapy, and this may precede clinical manifestations [44–48]. The knowledge of these peculiar toxicities is of utmost importance, because it ensures their early recognition while requiring the exclusion of differentials, mainly of infectious or tumoural nature.

#### Gastrointestinal, liver, and pancreatic toxicities

**Fig. 5:**
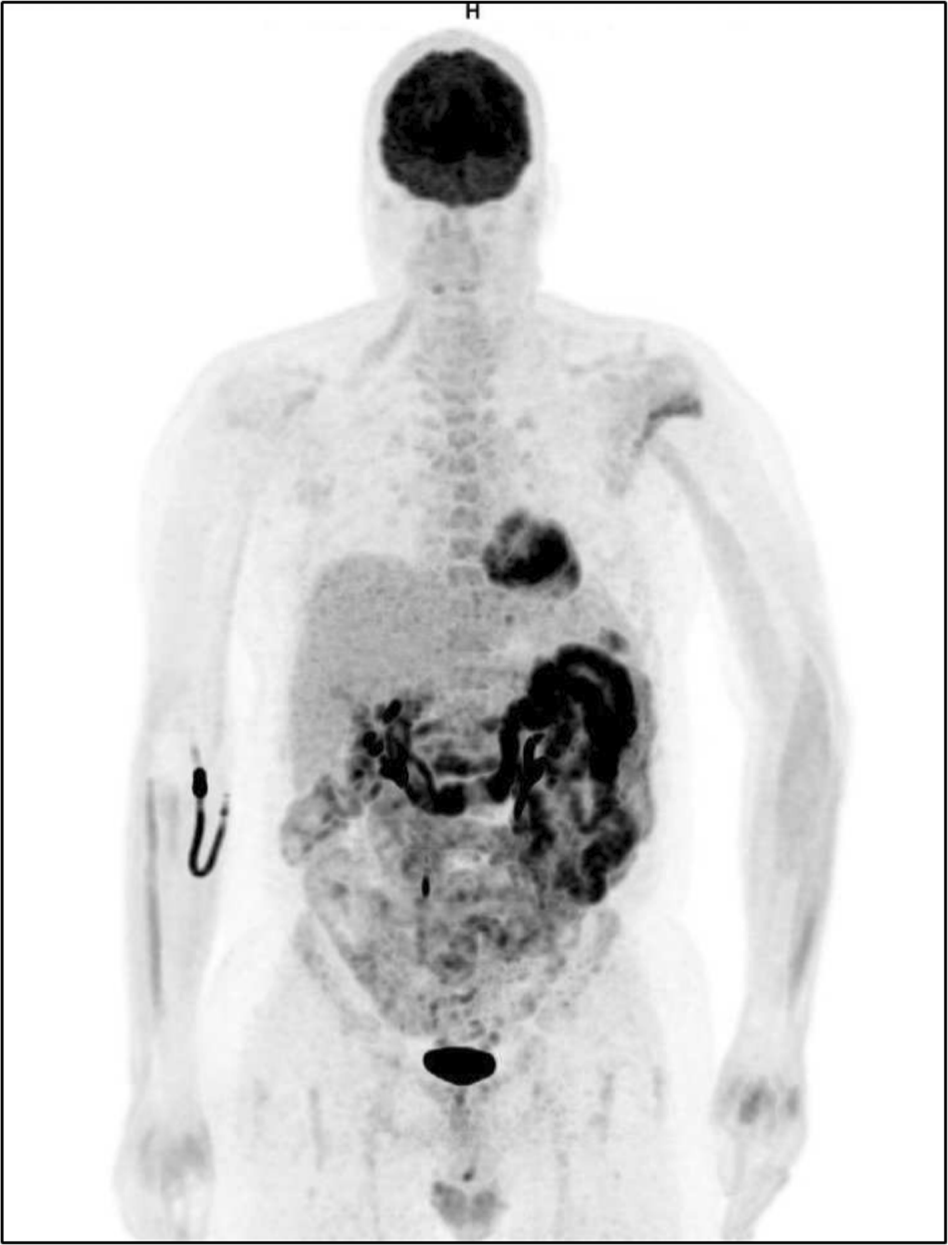
^18^F-FDG PET/CT image of a stage IV enterocolitis during anti-CTLA-4 treatment in a patient with metastatic melanoma. Immunotherapy was interrupted, and a high-dose steroid therapy was started

Colitis is one of the most frequent and potentially severe irAEs induced by anti-CTLA4 [38, 39]. Diarrhoea is reported in up to 50% of patients; other presenting symptoms include abdominal pain, bloody stools, and fever [49]. Complications include perforation, sepsis, frank bleeding, or dehydration [49]. At computed tomography (CT), ICI-induced colitis appears as a diffuse inflammatory pattern characterised by wall thickening, mucosal hyperenhancement, mesenteric hyperaemia, and air-fluid levels [47, 50, 51]. Segmental involvement has also been reported. At positron emission tomography (PET)/CT, colitis results in diffuse and intense 18F-fluorodeoxyglucose (^18^F-FDG)-avidity along the entire bowel [47, 50] (Fig. 5). These features may precede the onset of symptoms. Colonoscopy may show erythaema, ulceration, and mucosal friability, whereas neutrophilic, lymphocytic, or eosinophilic intraepithelial infiltrates and crypt invasion can be found on pathologic specimens [49, 50].

ICI-related hepatitis has been reported in up to 19% of patients receiving anti-CTLA4, whereas it is rarely reported when using anti-PD1 or anti-PDL1 [40, 42]. Although it is usually limited to a mild elevation of transaminases, life-threatening liver dysfunction may occur [38, 39]. Imaging findings are shared with other causes of acute liver dysfunction, ranging from the absence of detectable abnormalities to hepatomegaly with parenchymal heterogeneity, periportal oedema, and perihepatic ascites [47, 50, 51]. While physiological ^18^F-FDG uptake is usually not affected by diffuse liver diseases, a case of ICI-induced hepatitis showing an intense liver ^18^F-FDG-uptake area at PET/CT has been reported [52].

The pancreas is rarely affected by irAEs, resulting in elevation of amylase and lipase or hyperglycaemia and diabetes [38, 39, 41]. Symptomatic pancreatitis is very rare. Classic features of acute pancreatitis including enlargement and oedema of the pancreatic gland associated with peripancreatic oedema and fluid collections can be observed [50]. Diffuse pancreatic FDG uptake on positron emission tomography/CT has been reported [53].

#### Endocrine toxicities

Hypophysitis may develop during anti-CTLA4 treatment in up to 13% of patients, whereas it is rarely associated with anti-PD1 and anti-PDL1 ICI treatments [39]. Clinical and radiological features are similar to lymphocytic hypophysitis. Fatigue, headache, and hypopituitarism-related symptoms are often reported. Hypophysitis can incidentally be detected by imaging in asymptomatic patients or before onset of symptoms. It presents as an enlarged hypophysis on brain CT or magnetic resonance imaging (MRI), or an ^18^F-FDG-avid pituitary gland on PET/CT [46, 54, 55]. Any suspected hypophysitis requires an MRI of the pituitary gland to confirm the diagnosis and exclude mimickers such as metastasis and pituitary adenoma. On MRI, the pituitary gland is enlarged without mass effect on the optic chiasma, with a thickening of the infundibulum, and the hyperintensity of the posterior part of the gland is often missing [56, 57]. There is also a homogeneous or heterogeneous enhancement of the pituitary gland on contrast-enhanced images [56, 57].

Generally asymptomatic, ICI-induced thyroid dysfunction often presents as mild hypothyroidism or hyperthyroidism on blood tests, with detectable anti-thyroid peroxidase and anti-thyroglobulin antibodies in most cases [58]. It is more frequently reported in patients receiving combined immunotherapy (20%) than in those with anti-PD1/antiPD-L1 (up to 10%) or anti-CTLA4 (around 5%) monotherapy [38, 39]. Ultrasound is the tool of choice in this setting. Thyroid enlargement with heterogeneous and hypoechoic parenchyma, often with a nodular or pseudonodular pattern, may be observed [55]. An increased vascularity at colour-Doppler evaluation may be also found. Nevertheless, it may be incidentally detected as diffuse hypermetabolic thyroid gland at restaging ^18^F-FDG PET/CT [47, 55].

Rarely, primary or secondary adrenalitis may occur during immunotherapy, leading to adrenal insufficiency [38, 39]. The adrenal glands show bilateral enlargement at conventional imaging and bilateral mild ^18^F-FDG-avidity on PET/CT [55, 59].

#### Thoracic and cardiac toxicities

Pneumonitis is an autoimmune toxicity with a wide range of clinical course, ranging from mild dyspnoea to life-threatening respiratory failure [60], with up to 2% of patients developing severe pneumonitis [38]. Unlike the majority of irAEs, it is less common with anti-CTLA-4 monotherapy than with anti-PD-1 treatment [38, 60]. It occurs in up to 5% of patients receiving anti-PD1 and anti-PDL1, while it arises in 10% of patients receiving combination treatment [38, 60, 61], with higher odds of pneumonitis in non-small cell lung cancer compared with melanoma [61–63]. It is noteworthy that after pneumonitis resolution, some patients may be able to restart PD-1 inhibitor therapy without experiencing recurrent pneumonitis. Nevertheless, recurrence may occur in this setting, and pneumonitis flare corresponding to a recurrence of the pneumonitis after the completion of a corticosteroid taper without restarting ICIs or any other systemic agents have also been described in some cases [64].

**Fig. 6:**
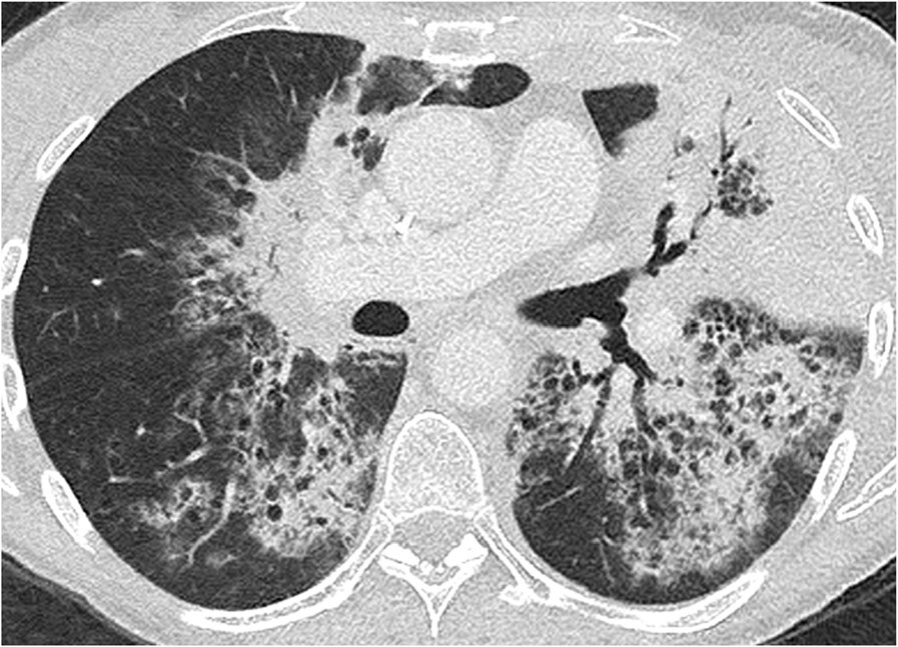
Immune-related pneumonitis presenting as an organising pneumonia pattern in a patient with metastatic lung cancer that occurred after 13 cycles of anti-PD1 therapy. This axial CT image in lung windowing shows multifocal alveolar consolidations in a subpleural and peribronchovascular location, predominating at the level of the left upper lobe. Although suggestive of a diagnosis of organising pneumonia, infectious or tumoural lesions were excluded by means of a brochoalveolar lavage. Note that numerous round lucencies are visible within the alveolar consolidations, corresponding to associated centrilobular emphysema in this heavy smoker patient

The spectrum of imaging aspects varies between minor interstitial anomalies up to acute interstitial pneumonia or acute respiratory distress syndrome pattern, these aspects reflecting pneumonitis grades [64]. It includes patterns of organising pneumonia, the most commonly seen, hypersensitivity pneumonitis, non-specific interstitial pneumonitis, or non-specified pneumonitis [64, 65]. Major elementary lesions are ground-glass opacities with a variable extent and location, reticulations, and alveolar consolidations, typically with a subpleural and peribronchovascular distribution in organising pneumonia pattern [61] (Fig. 6). Progression of an organising pneumonia pattern to a non-specific ground-glass opacity has been described. In addition, nodular aspect mimicking a tumour recurrence, as well as pembrolizumab-associated bronchiolitis, pleural effusions, or tracheitis, may be observed [45]. The diagnosis may be sensitive in case of underlying disease such as chronic obstructive pulmonary disease or previous radiotherapy. Moreover, if imaging appearances may suggest the diagnosis of immune-related pneumonitis, the establishment of the final diagnosis may remain challenging. The differential diagnosis must always be kept in mind, especially infectious disorders or tumour recurrence, this requiring a bronchoalveolar lavage in numerous cases of lung parenchymatous changes, if enabled by patient’s conditions.

**Fig. 7:**
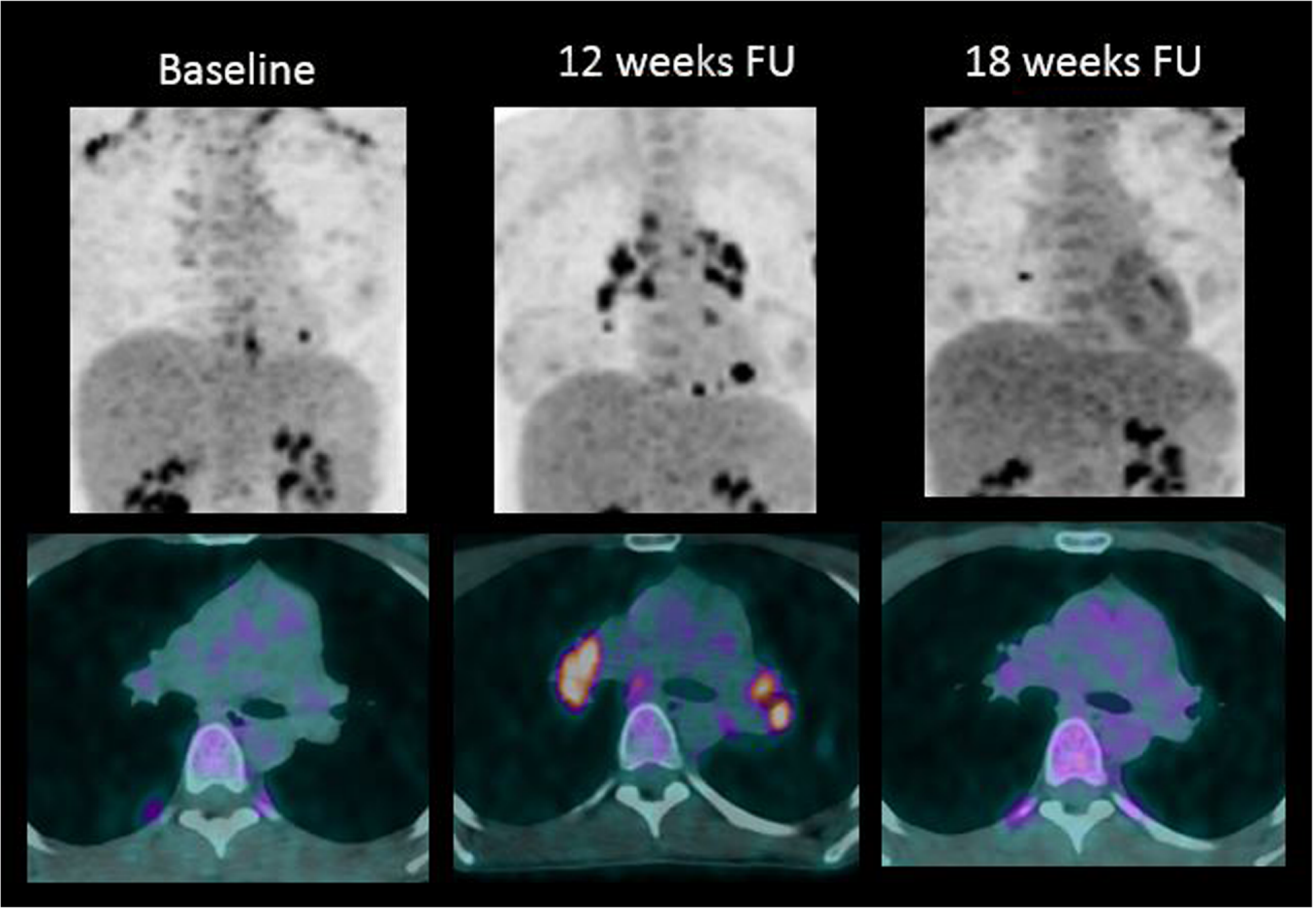
Sarcoid-like reactions in a patient with metastatic melanoma treated with ipilimumab (antiCTLA-4) and nivolumab (anti-PD-1). Twelve-week follow-up (FU) ^18^F-FDG PET/CT images show typical hypermetabolic symmetric mediastinal and hilar lymph node enlargement, very suggestive of a sarcoid-like reaction. These features disappeared on the following ^18^F-FDG-PET/CT images at 18-week FU, confirming the diagnosis of sarcoid-like reaction under immunotherapy

Sarcoid-like reactions, including lymphadenopathy and pulmonary granulomatosis, have been reported in up to 5–7% of patients treated with ICIs [43, 47]. Typical imaging findings are symmetric mediastinal and hilar lymph node enlargement, which may appear hypermetabolic on PET/CT (Fig. 7). Micronodules with perilymphatic distribution, especially in subpleural location, may also be seen [66].

Rarely, ICIs may lead to cardiac toxicity, including myocarditis, arrhythmias, Takotsubo cardiomyopathy, and pericarditis. The incidence of myocarditis range from 0.1 to 1%, and a fulminant course is common with fatal case rates of 25–50% of them [38, 39]. Most cases occur shortly after initiation of ICI therapy. Symptoms may vary, ranging from sudden onset of shortness of breath, chest pain, to heart failure. Importantly, a normal electrocardiogram, biomarkers, or a preserved left ventricular function do not rule out ICI-associated myocarditis. There is an undeniable role of cardiac MRI that can show characteristic findings of acute myocarditis, including myocardial oedema and late gadolinium enhancement in the focal subepicardial lateral wall [67, 68]. Cases of pericarditis, sometimes fatal, have also been reported [69].

#### Other toxicities

While arthralgias, myalgia, and inflammatory arthritis frequently occur as irAEs, vasculitis is an uncommon presentation, involving large vessels, nervous system, and less commonly medium and small vessels [70]. ICI-associated myositis with or without myasthenia gravis are the more frequent neuromuscular complications [71].

### How to predict response to immunotherapy

Remarkable responses to immunotherapies are currently limited to a minority of patients and indications. This highlights the need to identify more effective biomarkers that can be used in clinical routine, not only for an appropriate patient selection but also to offer personalised therapy.

The most extensively studied biomarker is the PD-L1 status. For example, in patients with newly diagnosed advanced non-small cell lung cancers and ≥ 50% PD-L1 expression, the combination of chemotherapy plus anti-PD-1 treatment with pembrolizumab significantly improved their objective response rate, progression-free survival, and overall survival [72]. However, its clinical use has been hampered by its dynamic expression that changes in relation to local cytokines and other factors. Thus, the threshold that separates a *positive* from *negative* PD-L1 expression remains debated and varies for each tumour type. Based on this experience, at present, no patient with advanced cancer and an established clinical rationale for the use of ICIs should be refused on the basis of lack of PD-L1 expression.

The rate of somatic mutations in tumour, the so-called tumour mutational burden (TMB), has also shown a potential response to ICIs. Mutated proteins can be recognised as non-self neo-antigens more easily by the adaptive immune system. In this context, ICIs are more effective. However, the TMB cutoff that could predict a response to ICI for each tumour type has shown to be variable [73]. No association was found between high TMB and survival in patients not treated with ICI, underlying the predictive value of high TMB for ICI therapies [74].

**Fig. 8:**
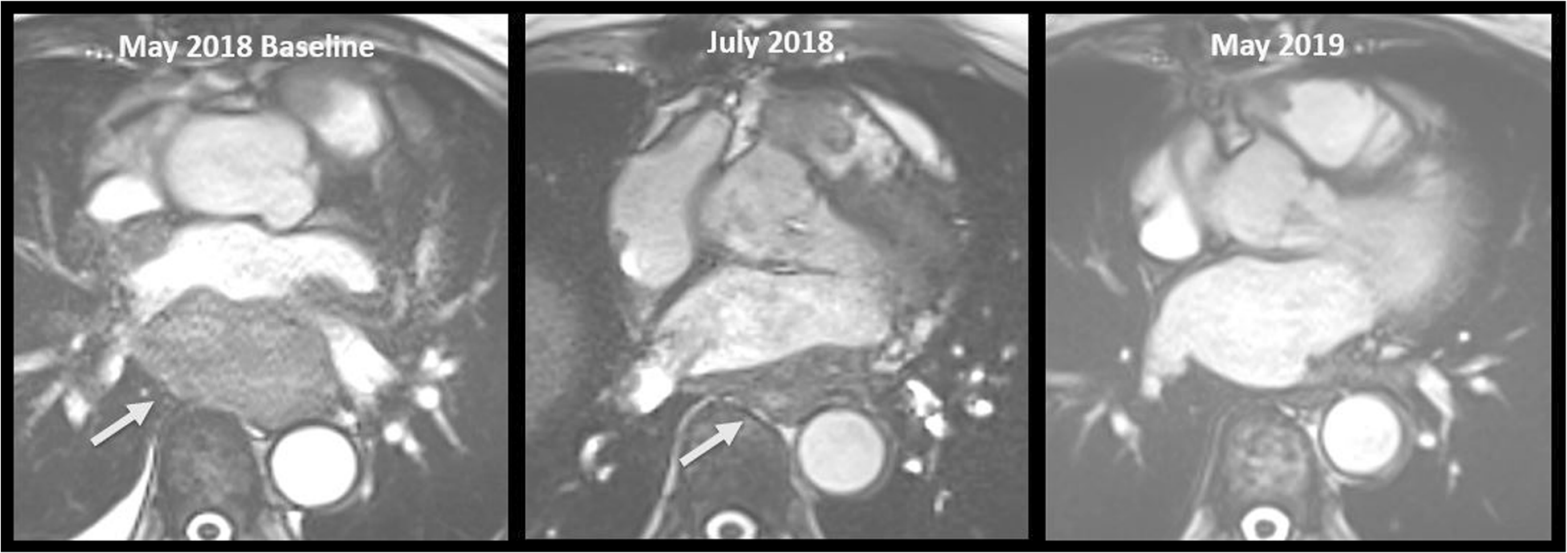
A 75-year-old woman with a low-differentiated primary cardiac sarcoma with microsatellite instability, treated with pembrolizumab (anti-PD-1). Baseline contrast-enhanced MRI image shows a large retroatrial mass (arrow). Two months follow-up (FU) imaging shows a good reduction in the size of the mass assessed as -34% according to iRECIST criteria (partial response, arrow). One year FU imaging shows a complete response

The mismatch repair proteins play a crucial role in the repair of DNA sequence mismatches during replication. A defective mismatch repair system leads to errors in DNA replication that accumulate in microsatellites, resulting in microsatellite instability. These defects are a result of either a germline mutation in the mismatch repair gene (Lynch syndrome) or more commonly as epigenetic inactivation of them. Tumours that are classified to have high microsatellite instability also have an accumulation of somatic mutations, resulting in a higher neoantigen load, which promotes activation and recruitment of T cells and hence sensitivity to immunotherapy [75] (Fig. 8). Currently, the National Comprehensive Cancer Network guidelines encourage microsatellite instability testing for all patients with advanced gastrointestinal cancer, and for this population, ICIs are FDA-approved. Microsatellite instability is highly variable among cancers, being most common in gastric and colorectal cancers (11.1%), whereas other tumour types such as pancreatic cancer are low [76].

Tumour infiltrating lymphocytes correspond to lymphocytes that directly oppose or surround tumour cells. The percentage degree of TILs has been shown to correlate with a favourable prognosis in several tumours including melanoma and breast and ovarian cancers [77, 78]. The degree of TILs may be defined by both the extent and density of the TILs using an “immunoscore” based on the numeration of CD3+ and CD8+ T cell at the intratumoural region as well as at the invasive margin area [79]. Finally, three different immune profiles have been described: (1) the *immune inflamed* with dense CD8+ T cell infiltration within the tumour (highest probability of response), (2) the *immune-excluded* with abundant immune cells around the tumour but not penetrating inside the tumour (intermediate probability of response), and (3) the *immune desert* with few or no CD8+ T cells (lowest probability of response to immunotherapy).

Only few imaging biomarkers have been studied for predicting response to immunotherapy. A radiomics approach was used to assess tumour-infiltrating CD8+ T cells in patients included in phase 1 trials of PD-1 and PD-L1 monotherapy [80]. A radiomic signature of CD8+ T cells was developed using CT images and RNA sequencing data from 135 patients (training set) and validated on three different cohorts of patients including 137 patients treated with anti-PD-1 and anti-PD-L1 drugs. High baseline radiomics score was associated with higher proportion of patients with objective response at 3 and 6 months. Moreover, high radiomics score was significantly associated with improved outcomes with a median overall survival of 24.3 *versus* 11.5 months in high and low radiomics score, respectively.

Molecular imaging techniques using radioactive tracers that target PD-1 and PD-L1 have also been explored in a preclinical study [81, 82]. A first-in-human study using PET with ^89^Zr-labeled atezolizumab has been conducted in 22 patients to predict response to PD-L1 treatment [83]. The authors showed a significant correlation between ^89^Zr-labeled atezolizumab uptake and patient outcomes in terms of progression-free and overall survival. Interestingly, responses were better correlated with baseline PET tracer fixation than with PD-L1 status using immunohistochemistry or RNA sequencing of post-tracer biopsies.

In this context, the potential applicability of these biomarkers in different disease settings is still pending with probably the exception of first-line lung cancer for PD-L1 expression.

## Conclusions

Cancer immunotherapy is becoming, in a few years, one of the most promising treatments of wide types of cancers. Currently, immunotherapy benefits only to some patients, and selecting patients who will benefit from immunotherapy is one of the major future challenges. Familiarity with the specificity of response and immune-related side effects is essential for radiologists to accurately evaluate the response to treatment and help clinician for optimal patient management. Although pseudoprogression occurs only in few patients treated with ICIs, new criteria (irRC, irRECIST, iRECIST, and imRECIST) has been developed to address this issue in clinical trials. Their use in clinical routine should be prudent as data are still limited. Moreover, new image interpretation challenges will probably occur in the future with the increasing use of combined therapies with conventional chemotherapy and locoregional therapies (radiotherapy, cryotherapy, etc.) as well as other types of immunotherapy such as vaccines or adoptive cell transfer therapy.

## Data Availability

Not applicable

## Abbreviations

^18^F-FDG: 18F-fluorodeoxyglucose
CT: Computed tomography
CTLA4: Cytotoxic T-lymphocyte antigen 4
DNA: Deoxyribonucleic acid
FDA: Food and Drug Administration
ICI: Immune checkpoint inhibitor
imRECIST: Immune-modified RECIST
irAEs: Immunotherapy-related adverse events
irRC: Immune-related response criteria
irRECIST: Immune-related RECIST
MRI: Magnetic resonance imaging
PD-1: Programmed cell death protein 1
PD-L1: Programmed cell death protein ligand 1
PET/CT: Positron emission tomography/computed tomography
RECIST: Response evaluation criteria in solid tumors
RNA: Ribonucleic acid
TILs: Tumour-infiltrating lymphocytes
TMB: Tumour mutational burden
